# Docosahexaenoic Acid-Rich Fish Oil Supplementation Reduces Kinase Associated with Insulin Resistance in Overweight and Obese Midlife Adults

**DOI:** 10.3390/nu12061612

**Published:** 2020-05-30

**Authors:** Rohith N. Thota, Jessica I. Rosato, Tracy L. Burrows, Cintia B. Dias, Kylie A. Abbott, Ralph N. Martins, Manohar L. Garg

**Affiliations:** 1 Nutraceuticals Research Program, School of Biomedical Sciences & Pharmacy, University of Newcastle, Callaghan NSW 2308, Australia; R.thota@massey.ac.nz (R.N.T.); jessica.rosato@uon.edu.au (J.I.R.); Cintia.botelhodias@mq.edu.au (C.B.D.); kylie.abbott@uon.edu.au (K.A.A.); 2 Riddet Institute, Massey University, Palmerston North 4474, New Zealand; 3 School of Health Sciences, University of Newcastle, Callaghan NSW 2308, Australia; tracy.burrows@newcastle.edu.au; 4 Department of Biomedical Sciences, Macquarie University, North Ryde NSW 2109, Australia; ralph.martins@mq.edu.au; 5 School of Medical Sciences, Edith Cowan University, Joondalup WA 6027, Australia

**Keywords:** long-chain omega-3 polyunsaturated fatty acids, docosahexaenoic acid, insulin resistance, obesity, Alzheimer’s disease, glycogen synthase kinase-3

## Abstract

Targeting kinases linked to insulin resistance (IR) and inflammation may help in reducing the risk of type 2 diabetes (T2D) and Alzheimer’s disease (AD) in its early stages. This study aimed to determine whether DHA-rich fish oil supplementation reduces glycogen synthase kinase (GSK-3), which is linked to both IR and AD. Baseline and post-intervention plasma samples from 58 adults with abdominal obesity (Age: 51.7 ± 1.7 years, BMI: 31.9 ± 0.8 kg/m^2^) were analysed for outcome measures. Participants were allocated to 2 g DHA-rich fish oil capsules (860 mg DHA + 120 mg EPA) (n = 31) or placebo capsules (n = 27) per day for 12 weeks. Compared to placebo, DHA-rich fish oil significantly reduced GSK-3β by −2.3 ± 0.3 ng/mL. An inverse correlation (*p* < 0.05) was found between baseline insulin and IR and their changes following intervention only in participants with C-reactive protein levels higher than 2.4 mg/L. DHA-rich fish oil reduces GSK-3 and IR, suggesting a potential role of long-chain omega-3 polyunsaturated fatty acids (LCn-3PUFA) in ameliorating AD risk.

## 1. Introduction

Obesity is now a major global epidemic [1]. It not only predisposes to an array of risk factors such as insulin resistance (IR) and chronic low-grade inflammation but also poses a risk to several non-communicable chronic diseases like type 2 diabetes (T2D) and cardiovascular disease [2], leading to increased morbidity and mortality amongst adults. The vascular effects of obesity may have a role in the development of a rapidly expanding disease in the elderly population, Alzheimer’s disease (AD) [3]. Although the mechanisms are not precise, the detrimental impact of obesity on cognitive function may be, at least in part, due to vascular defects like IR and chronic low-grade inflammation, impaired insulin metabolism or insulin resistance [4]. There are multiple risk factors for AD, including age, obesity, chronic inflammation, genetics and insulin resistance [3]. A prospective study of >10,000 participants examining the association between body mass index (BMI) and dementia over 36 years reported that obese participants (BMI ≥ 30) at midlife had more than 3-fold increase in the risk of developing AD compared to those with a normal weight range (18.5–25 kg/m^2^) [5]. Blood-based kinases such as protein kinase R and c-Jun N-terminal kinase, and in particular glycogen synthase kinase-3 (GSK-3) implicated in both IR and Tau phosphorylation and Aβ plaques, were found to be elevated in individuals with mild cognitive impairment and AD [6]. Interventions targeting such kinases in the high-risk groups might be beneficial as an early intervention to reduce the risk of T2D and AD.

Research studies on long-chain omega-3 polyunsaturated fatty acids (LCn-3PUFA), eicosapentaenoic (EPA; 20:5n-3) and docosahexaenoic acid (DHA; 22:6n-3) have previously shown neuroprotective properties [7,8]. A substantial amount of evidence is available on the effects of LCn-3PUFA, specific to DHA and EPA, over neuronal membrane properties, brain plasticity, inflammation and memory [9]. DHA is quantitatively the most important omega-3 PUFA in the brain; however, the endogenous synthesis of LCn-3PUFA is low within the brain compared with uptake from the plasma pools [10], thus suggesting that the brain maintains DHA levels via the uptake from dietary sources such as fish or LCn-3PUFA oils (fish or algae oils). At a cellular level, EPA and DHA have been shown to contribute to the modulation of gene expression and of kinases, as well as activation of signaling pathways and metabolite formation, involved in neuroprotection [11].

LCn-3PUFA may play a role in the alleviation of obesity-induced IR by promoting the adipose tissue function and maintaining its integrity [12,13]. Strong evidence from pre-clinical and ex vivo studies suggests mechanisms of EPA and DHA in ameliorating IR linked to obesity [14]. Epidemiological evidence presents an inverse association between higher levels of circulating LCn-3PUFA and the incidence of type 2 diabetes [15,16]. Cross-sectional studies [17] and a meta-analysis [18] from our research team have reported an inverse sex-dependent relationship between LCn-3PUFA, IR and type 2 diabetes, demonstrating favorable effects, only in females. These results suggest that sex may also be one of the potential confounders contributing to the inconsistent findings between LCn-3PUFA and diabetes. Interestingly, clinical trials have produced contrasting results to that of in vivo evidence from animal studies [19]. Therefore, further research is required to elucidate the role of LCn-3PUFA in ameliorating IR.

In the current study, we aimed to determine the effect of fish oil enriched with DHA as a source of LCn-3PUFA, on GSK-3 and IR in a randomized controlled trial involving overweight and obese individuals.

## 2. Materials and Methods

### 2.1. Participants

Participants were recruited for the ‘Do omega-3 polyunsaturated fatty acids have a gender-specific effect on insulin resistance?’ double-blind randomized controlled trial (ACTRN: 12616000287437) from the Newcastle (Callaghan, NSW, Australia) community through advertisements, local media and social media, and via the Hunter Medical Research Institute Volunteer Register. Volunteers were eligible to participate if they were aged between 18 and 70 years, had a body mass index (BMI) between 25–45 kg/m^2^, had a waist circumference ≥88 cm (females) or ≥102 cm (males). Participants were excluded if they were diagnosed with diabetes, were taking medications to lower blood sugar levels or to influence insulin sensitivity (e.g., Metformin), if they were pregnant or breastfeeding, were intolerant to the study products, were taking anticoagulants such as aspirin or warfarin, had a history of severe gastrointestinal or neurological disorders, consumed >2 serves of oily fish per week or reported taking fish oil supplements.

### 2.2. Study Design and Intervention

Eligible participants were randomly allocated using a computer-generated sequence to one of two groups, DHA-rich fish oil or Placebo (corn oil). Participants were asked to consume either 2 × 1 g DHA-rich fish oil capsules/day containing 430 mg DHA + 60 mg EPA or 2× placebo capsules each containing 1 g of corn oil, for a period of 12 weeks. DHA-rich fish oil and placebo capsules were manufactured and provided in kind by EPAX, Norway. The dose of DHA was based on the previous studies that showed efficacy of DHA on IR [20,21]. Participants were asked to maintain their habitual diet and usual physical activity levels for the study duration, with dietary intake (3-day food record) and physical activity (international physical activity questionnaire, IPAQ—long form questionnaire) measured at the beginning and end of the study period to ensure compliance. Compliance to the study intervention was measured using capsule log, return capsule count, and gas chromatography analysis of the fatty acid profile of erythrocyte membranes. The study was conducted in accordance to the Declaration of Helsinki and following Good Clinical Practice guidelines and had ethical approval from the University of Newcastle Human Research Ethics Committee (H-2015-0167). Written informed consent was obtained from all participants prior to enrolment in the study.

### 2.3. Outcome Measurements

All anthropometric measurements were conducted using bio-electrical impedance scales (InBody 230, Biospace Co., Ltd., Seoul, Korea), according to standard operating procedures. Participants’ height (cm) was measured using a wall-mounted stadiometer (SE206, Seca, Hamburg, Germany) and waist circumference (WC in cm) was measured using a non-tensible tape measure at the mid-point between the lower rib and the iliac crest. Body mass index (BMI; kg/m^2^) was calculated using the formula BMI = weight (kg)/height (m^2^). A 20 mL fasting venous blood samples were collected from the antecubital vein into pre-coated vacutainers for plasma glucose (mmol/L), serum insulin (μIU/L), high sensitivity C-reactive protein (CRP; mg/L) and total cholesterol (TC; mmol/L), and were analysed by a commercial pathology laboratory (Hunter New England Area Pathology Services). Erythrocyte fatty acid composition was analysed using a one-step transmethylation method followed by gas chromatography (Hewlett Packard 7890A Series GC with Chemstations Version A.04.02) [22]. GSK-3β was analysed using enzyme-linked immunosorbent assay (ELISA) kits. This ELISA kit has high specificity for human GSK-3β and no detectable cross-reactivity with other proteins which might interfere with the assay process and results (manufacturer of the kit- Aviva systems biology, detection range: 0.625–40 ng/mL; mean intra-assay CV < 10%; mean inter-assay CV < 12%). HOMA-IR, used as an indicator of insulin resistance, was calculated using an established equation—fasting glucose (mmol/L) * fasting insulin (μIU/L)/22.5.

### 2.4. Statistical Analysis

A total of seventy-two participants were recruited for the main study. Required plasma samples were available for fifty-eight participants for the analysis of the current study outcomes. For this study, n = 58 (27 allocated to placebo group and 31 allocated to DHA-rich fish oil intervention group) were analysed for GSK-3β resulting in a study power of 98.9% for the detection of a 2.3 ng/mL reduction in serum GSK-3β level (1.8 ng/mL SD, alpha value set at 0.01). A power calculation was conducted using PS power and sample size calculator (Version 3.1.2, 2014). Data were tested for normality using Shapiro–Wilk’s test and histogram, and data described as the mean ± standard deviation (SD) or median (interquartile range; IQR) as appropriate. Effect sizes are presented as the mean difference [95% Confidence Interval; 95% CI]. Changes in the outcome measures within the placebo and the intervention group across the study period were assessed using paired *t*-test (parametric) or Wilcoxin Sign-rank test for non-parametric data. Treatment effects were assessed using regression to assess absolute change from baseline using the placebo group as a reference and adjusting for baseline levels. Pearson’s product–moment correlations with Bonferroni correction were used to assess linear relationships between continuous variables, with between-group differences assessed using the immediate command *cortesti* in Stata to test the equality of two correlation coefficients in independent samples. It considers the respective correlation co-efficient (i.e., *r*-value) and sample size, with *p* < 0.05 signifying a significant difference between correlations. Multiple regression models were used to investigate effects of baseline factors (age, sex, BMI, and physical activity) on results. Subgroup analysis on effects of intervention were carried out in people with high and low baseline CRP levels. All statistical tests were two tailed, and alpha was set at *p* < 0.05. All statistical analyses were conducted using Stata (Version 14.2, StataCorp LLC, Lakeway, TX, USA).

## 3. Results

### 3.1. Baseline Characteristics

A total of 58 participants (DHA-rich fish oil n = 31; placebo n = 27) were analysed for GSK3β and included in the final analyses. Baseline characteristics of participants have been summarized in Table 1. Overall the participants both males and females were predominately middle aged (51.1 ± 1.7 years), and mild to moderately obese (BMI: 31.9 ± 0.8 kg/m^2^; WC: 102.2 ± 2.4 cm). The median fasting insulin levels (10.1 (6.1)) of the study participants indicate hyperinsulinemia, as the normal adult fasting insulin levels are <10 mIU/L. Baseline CRP levels were positively correlated (*r* = 0.3513, *p* < 0.05) with baseline fasting insulin levels, indicating a close relationship between systemic inflammation and hyperinsulinemia. There were no significant differences between groups at the baseline (Table 1).

### 3.2. Compliance to the Study Products and Changes in the Participants’ Erythrocyte Membrane Fatty Acid Composition

**Figure 1:**
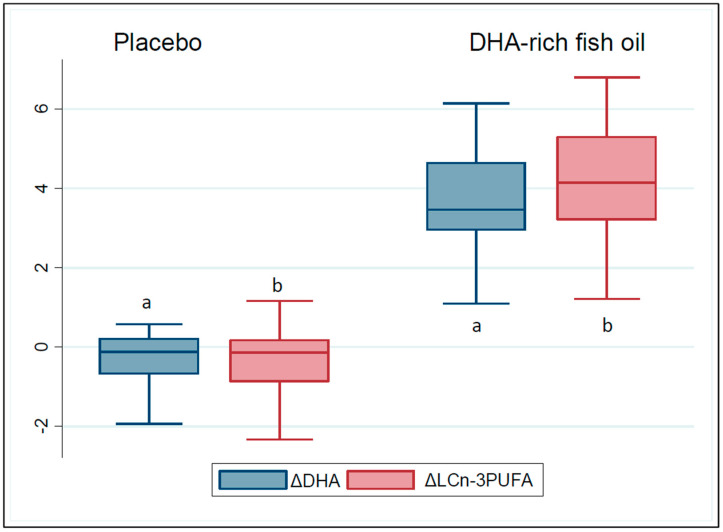
Change in erythrocyte membrane red blood cell (RBC) docosahexaenoic acid (DHA), and total long-chain omega-3 polyunsaturated fatty acids (LCn-3PUFA). Significance set at *p* < 0.05. Lowercase letters a and b represent significant difference between the placebo and the DHA-rich fish oil groups for LCn-3PUFA and DHA, respectively.

Compliance to the study capsule intake was high (>95%). Accordingly, the DHA-rich fish oil group showed an increase in DHA and LCn-3PUFA content in erythrocyte membranes compared to placebo (*p* < 0.001) (Figure 1), with increases in erythrocyte DHA (+3.5 (1.7) %*w/w*, *p* < 0.001), EPA (+0.5 ± 0.1%*w/w*, *p* < 0.001) and LCn-3PUFA (+4.1 (2.1) %*w/w*, *p* < 0.001) in the DHA-rich fish oil group, and no significant change in erythrocyte DHA (−0.1 (0.9) %*w/w*), EPA (−0.1 ± 0.1%*w/w*) and LCn-3PUFA (−0.1 (1.04)) in the placebo group (Table 2). There were no significant changes to dietary intake (kj) or physical activity levels across the intervention (data not presented), nor in anthropometric measurements in either FO or CO groups across the intervention period (*p* > 0.05 for all). Three participants in the FO group reported fishy burps, but there were no adverse events reported throughout the study.

### 3.3. Effect of DHA-Rich Fish Oil on GSK-3β

**Figure 2:**
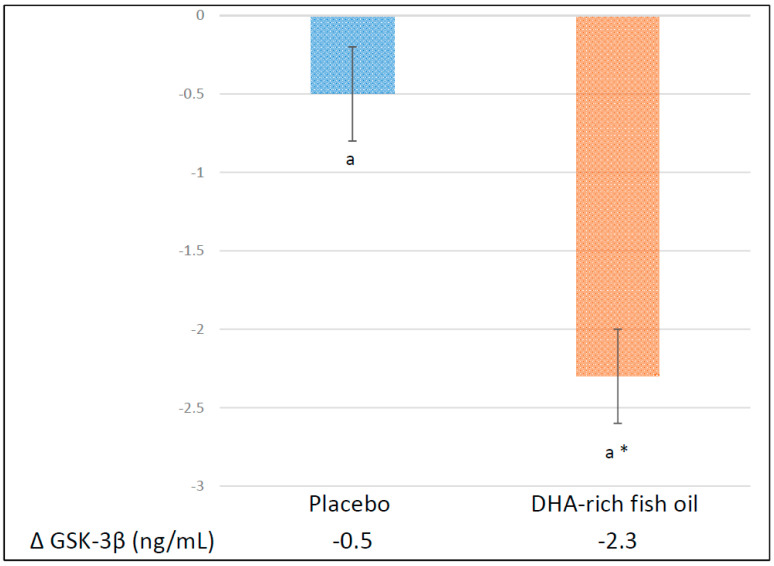
Absolute change in plasma GSK-3β from baseline to post-intervention in the placebo and the DHA-rich fish oil groups. Data are presented as the mean ± SEM. Significance is set at *p* < 0.05. * Significant difference in change within group. ^a^ A significant difference between the placebo and the DHA-rich fish oil groups. GSK-3β: glycogen synthase kinase-3β.

A 12 week DHA-rich fish oil supplementation significantly (*p* < 0.01) reduced GSK-3β levels (−2.3 ± 0.3 ng/mL) within that group and in comparison to the placebo group (*p* < 0.01) (Figure 2). Regression model indicated a significant treatment effect for GSK-3β (mean difference = −1.8 ng/mL [−2.7, −0.9], *p* < 0.05) across the course of the study (Table 2). The difference between groups remained significant for GSK-3β after adjusting for age, sex, BMI and baseline physical activity levels (adjusted R^2^ = 40.37, *p* < 0.05).

### 3.4. Effect of DHA-Rich Fish Oil on Fasting Insulin and Insulin Resistance

The DHA-rich fish oil group had a reduction in fasting insulin (−1.3 ± 0.7 mIU/L, *p* < 0.05) and HOMA-IR (−0.3 ± 0.2, *p* < 0.05), with no change observed in the CO group (*p* > 0.05) for insulin (0.4 ± 0.6) and HOMA-IR (0.1 ± 0.2). Regression models indicated a significant treatment effect for insulin (−1.7 (−3.5, 0.14), *p* < 0.05) and HOMA-IR (−0.4 (−0.9, 0.1), *p* < 0.05) (Table 2). The difference between groups remained significant (*p* < 0.05) for both insulin (adjusted R^2^—42.60) and HOMA-IR (adjusted R^2^—40.35) after adjusting for age, sex, BMI and baseline physical activity.

**Figure 3:**
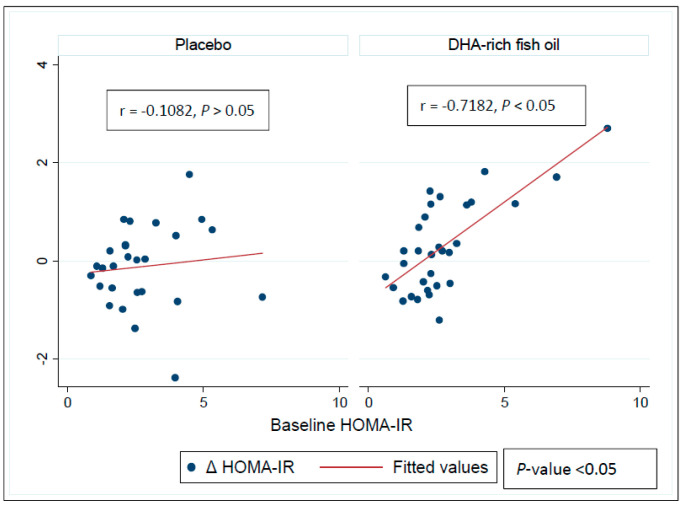
Pearson’s product–moment correlations exhibiting relationship between baseline HOMA-IR values and responses to intervention in the placebo and the DHA-rich fish oil groups. *p*-value represents differences in correlations between the groups. HOMA-IR: homeostatic model of assessment-insulin resistance.

**Figure 4:**
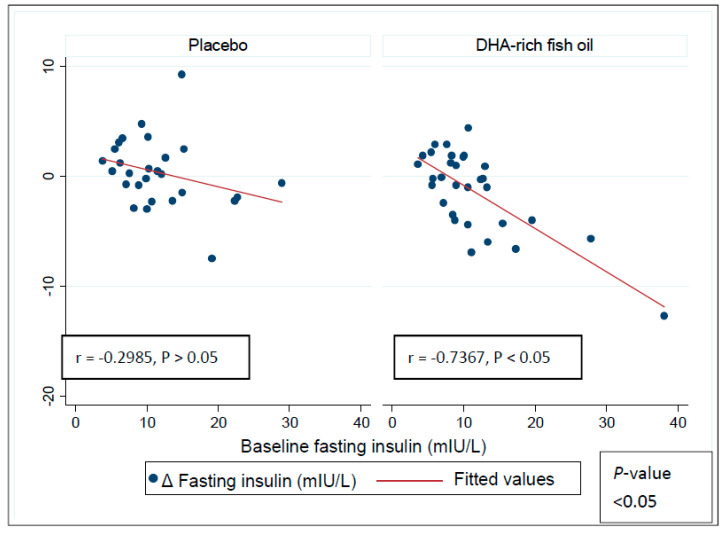
Pearson’s product–moment correlations exhibiting relationship between baseline fasting insulin values and responses to intervention in the placebo and the DHA-rich fish oil groups. *p*-value represents differences in correlations between the groups.

Pearson’s product–moment correlations indicated an inverse correlation between the baseline insulin (*r* = −0.7182, *p* < 0.05) and HOMA-IR (*r* = −0.7367, *p* < 0.05) levels and change in the HOMA-IR (Figure 3) and Insulin levels (Figure 4) in the DHA-rich fish oil group across the intervention period, with no significant correlation seen in the placebo group (insulin: *r* = −0.2985, *p* > 0.05) and HOMA-IR (*r* = −0.1082, *p* > 0.05). Further, these correlations differed between the DHA-rich fish oil and placebo groups (*p* < 0.05 and *p* < 0.05 for insulin and HOMA-IR respectively). A sensitivity analysis indicated no difference in significance changes for Insulin (*p* < 0.05) or for HOMA-IR (*p* < 0.05) after removing a potential outlier in the DHA-rich fish oil group.

### 3.5. Effect of DHA-Rich Fish Oil on the Other Biomarkers

There were no significant (*p* > 0.05) changes for fasting glucose and CRP within the DHA-rich fish oil (Δ fasting glucose 0.04 ± 0.1 mmol/L; ΔCRP −0.7 ± 0.6 mg/L) and the placebo groups (−0.04 ± 0.1 mmol/L; ΔCRP −0.5 ± 0.4) (Table 2). Regression models did not indicate any significant differences in fasting glucose and CRP levels between the means of the DHA-rich fish oil and the placebo group (Table 2).

### 3.6. Effect of DHA-Rich Fish Oil Over GSK-3β, Insulin and HOMA-IR in Subgroups Based on Baseline Systemic Inflammation Status

**Figure 5:**
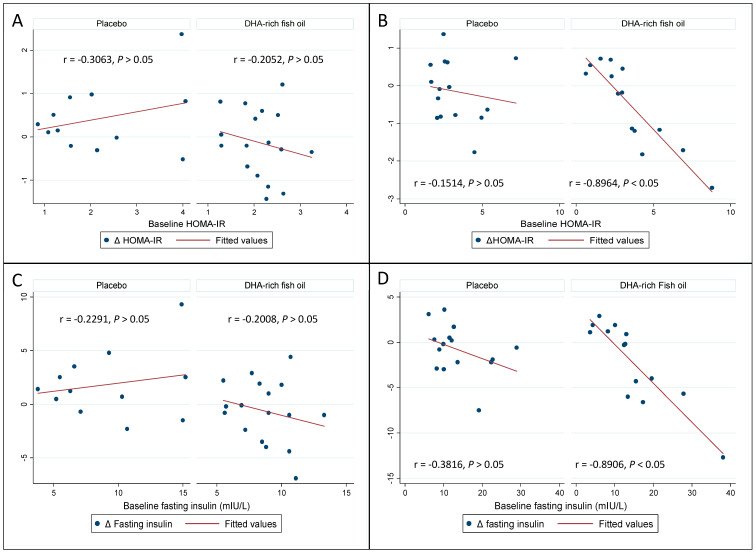
Pearson’s product–moment correlations exhibiting relationship between baseline HOMA-IR and fasting insulin values and responses to intervention in placebo and DHA-rich fish oil groups in study participants with C-reactive protein <2.4 mg/L (**A**,**C**) and ≥2.4 mg/L (**B**,**D**).

The study population was divided in two groups, categorising the study participants into groups above and below the median baseline CRP levels (2.4 mg/L). Summary of changes in the biochemical parameters of the participants between the groups is presented in Table 3. Pearson correlation analysis indicated a significant inverse correlation between the baseline insulin and HOMA-IR and change in insulin and HOMA-IR only in participants with CRP above 2.4 mg/L (Figure 5). These correlations were significant (*p* < 0.05) for both insulin and HOMA-IR between the CRP ≥2.4 mg/L and <2.4 mg/L groups. No significant differences were observed on the effect of DHA-rich fish oil over GSK-3β between the CRP ≥2.4 mg/L and <2.4 mg/L groups (*p* > 0.05).

## 4. Discussion

Dietary supplementation with DHA-rich fish oil was accompanied by a reduction in plasma levels of GSK3. DHA-rich fish oil also lowered IR and fasting insulin in individuals with abdominal obesity and hyperinsulinemia compared to the placebo group. The correlation analysis indicated a significant inverse relationship between fasting insulin and IR and changes in insulin and IR only in individuals with high baseline CRP values. No significant changes were observed in fasting glucose and CRP levels. In this study, baseline GSK-3β, insulin and IR were significant predictors of the response to the treatment. Therefore, a greater reduction in these parameters was observed in participants with higher baseline GSK-3β, IR and insulin values.

Hyperinsulinemia and IR are significantly associated with the development of both T2D and AD [23]. Cross-sectional studies have demonstrated significant associations of HOMA-IR with mild cognitive impairment and AD [24]. Presence of IR also accelerates the formation of neuritic plaques which are involved in the pathogenic process of AD [25]. A large body of pre-clinical evidence is currently available on the effects of LCn-3PUFA over insulin signalling and insulin sensitivity [14,26]. In line with these studies, epidemiological studies indicate a positive correlation between LCn-3PUFA status and insulin sensitivity [15]. However, systematic reviews and meta-analysis of clinical trials indicate ambiguous and inconclusive results over efficacy of LCn-3PUFA on insulin sensitivity and glycaemic control [14,19,27]. Results from this study suggest that these inconsistencies may be due to differences in the baseline levels of IR and insulin, as no benefit was apparent in participants with low levels of baseline IR or insulin.

GSK3 is widely expressed in two isoforms α and β. in the human tissues and can also be detected in the peripheral blood mononuclear cells [28]. GSK-3 is involved in the downstream signalling pathway of kinases (phosphatidylinositol 3-kinase) that are involved in insulin signalling and IR [29]. Dysregulation of insulin pathways, referred to as IR, is linked to increased levels of GSK3 [29]. GSK-3 induce IR via enhanced phosphorylation of insulin receptor substrate-1 protein, resulting in defective GLUT translocation interfering with insulin actions and glucose uptake [29]. Pre-clinical and clinical studies have shown that LCn-3PUFA improve insulin sensitivity by increasing insulin-stimulated disposal of amino acids and glucose and by improving insulin sensitivity [30,31]. In the current study, LCn-3PUFA was shown to reduce GSK-3 levels in individuals at high risk of IR and hyperinsulinemia. DHA-rich fish oil associated reduction in IR and fasting insulin levels suggests a possible GSK-3 mediated improvement in the insulin levels.

GSK3 is also implicated in the pathogenesis of AD via α-form linking to precursor tau phosphorylation and β-form linking to Aβ precursor protein [28]. Systematic reviews have concluded that dietary supplementation with LCn-3PUFA has the potential to ameliorate AD-related symptomology [8]. The current study supports previous findings that LCn-3PUFA supplementation may interfere with GSK-3β activity. Further studies are required to substantiate the efficacy of LCn-3PUFA on reducing GSK3 in peripheral blood mononuclear cells obtained from individuals with cognitive impairment or with a high risk of AD.

In addition, our study has also indicated that DHA-rich fish oil-associated reductions in IR and insulin levels were only significant in participants with high baseline CRP levels. As the previous evidence also strongly links inflammation and insulin resistance [32], these two observations may be an important consideration in assessing the efficacy of DHA and EPA on IR. Results from this study are also consistent with a systematic review, which showed that LCn-3PUFA supplementation reduced IR in populations who displayed at least one symptom of metabolic disorder, while there was no effect seen in healthy populations [27]. Our study population displayed at least one characteristic of metabolic dysfunction (i.e., either hyperinsulinemia or glucose levels in the range of impaired fasting glucose or abnormal CRP levels) and are either overweight or obese. The results in the present study indicate a narrow window for optimal efficacy of LCn-3PUFA on measures of IR when hyperinsulinemia and high inflammation are present before the onset of diabetes or AD. Targeting this window of opportunity may help optimize nutritional strategies aimed towards the reduction in IR and prevention of AD.

In this intervention, we used a DHA-enriched FO capsule. EPA and DHA have been shown to exhibit specific cellular and physiological functions. As mentioned above, DHA is more involved in the regulation of GSK3 than EPA. Further, DHA is more effective than EPA in stimulating peroxisome-proliferated activator receptor-gamma (PPAR-γ) and secretion of adiponectin in vitro; both of these are proposed mechanisms for the insulin-sensitising action of LCn-3PUFA [33]. Furthermore, administration of DHA derived lipid mediators (D-series resolvins) in knockout-mice models of diabetes reduced glucose intolerance and macrophage infiltration in adipose tissue [34]. Taken together, these suggest that DHA may be more effective than EPA in reducing IR.

In an intervention study with a similar intervention period (12 weeks), Browning et al. showed that LCn-3PUFA (1.3 g/day EPA + 2.6 g/day DHA) reduced IR in overweight women with a raised inflammatory profile at baseline, while no effect was observed in women with low levels of inflammation [35]. These observations are consistent with our findings that baseline levels of inflammation may influence the response of LCn-3PUFA on insulin and IR levels. GSK-3-mediated kinase pathways are implicated in regulating the host IR and inflammatory response, playing a pivotal role in regulating the pro- and anti-inflammatory cytokines [36]. In-vitro studies have shown inhibition of GSK-3, which may promote tolerance to inflammatory stimuli and suppress cytokine production [37]. Abnormal function of GSK-3 is already indicated in high inflammatory conditions such as AD, diabetes, and cancer [38]. Therefore, LCn-3PUFA-mediated reduction or lowering of the activity of GSK-3 could potentially explain the lowering in IR, and beneficial effects of LCn-3PUFA in people with high inflammation levels. Further research is warranted to delineate differential effects of EPA and DHA-rich formulations over these metabolic and inflammation parameters. In line with the observations from a meta-analysis with 68 randomized controlled trials [39] and systematic reviews [40], this study failed to report a significant effect of LCn-3PUFA on CRP.

Both log-based capsule count and fatty acid profiling exhibited a high level of compliance by participants in both the DHA-rich fish oil and the placebo groups. GSK-3β ELISA kits used to measure the primary outcome have high specificity for human GSK-3β and no detectable cross-reactivity with other relevant proteins that could potentially interfere with assay process. The study was adequately powered for GSK3β. The link between AD and IR is a relatively new and emerging area of research. In this study, we were able to provide basis for DHA-rich fish oil for use as a potential adjuvant for ameliorating common risk factors in early AD prevention interventions.

Though adequately powered, the results of this study remain a secondary analysis and are limited by their preliminary nature. A follow-up study with a narrow age and BMI range is required to substantiate effects of DHA-rich fish oil on GSK3β and determine whether it can affect neurological and metabolic parameters specific to T2D and AD. The preliminary results of this study may not be generalizable nor transferable to other populations. In relation to IR, we employed a surrogate marker (HOMA-IR) instead of a clamp technique, the gold-standard measure of IR. This may also be considered a limitation of the current study; however, HOMA-IR has been shown to have a relatively high correlation (R~0.77) with clamp technique and is currently the most used surrogate marker to measure longitudinal changes to IR [41]. Therefore, long-term studies, stratified for inflammation, may be required for substantiating the results from the current study.

## 5. Conclusions

DHA-rich fish oil supplementation significantly reduced the GSK-3β levels in individuals with obesity, a key kinase that is linked to the pathogenesis of T2D and AD. This suggests that fish oil enriched in DHA may be a potential early intervention option to reduce the chances of developing metabolic disease in high-risk individuals. DHA-enriched fish oil supplementation also significantly reduced fasting insulin levels and HOMA-IR compared to placebo. LCn-3PUFA-mediated reduction in the GSK-3 levels could be a potential mechanism involved in reducing the IR. Higher levels of insulin and HOMA-IR at baseline were associated with a greater reduction in insulin and IR in the DHA-rich fish oil-receiving group over the treatment period. Further, subgroup analysis indicated a significant reduction in insulin and HOMA-IR values with high baseline systemic inflammation compared to those with lower systemic inflammation. Thus, DHA-enriched fish may be effective in reducing insulin resistance in populations with higher inflammation levels. Overall, DHA-rich fish oil supplementation may be a beneficial adjunct therapy to dietary and lifestyle advice for overweight and obese men and women to reduce the risk of associated comorbidities.

## Figures and Tables

**Table 1:** Baseline characteristics of the study participants.

Characteristics	Total	Placebo	DHA-Rich
			Fish Oil
	(n = 58)	(n = 27)	(n = 31)
Age (years)	51.1 ± 1.7	48.8 ± 2.6	53.2 ± 2.5
Males/females (n/n)	20/38	9/19	11/19
Anthropometry measures			
Body weight (kg)	89.4 ± 2.4	92.8 ± 4.1	86.3 ± 2.8
Muscle mass (kg)	31.5 ± 1.2	31.7 ± 1.2	31.4 ± 2.1
Fat free mass (kg)	55.2 ± 1.4	56.3 ± 2.0	54.3 ± 2.1
Body mass index (kg·m^−2^)	31.9 ± 0.8	33.5 ± 1.5	30.6 ± 0.8
Waist circumference(cm)	102.2 ± 2.4	106.0 ± 3.9	104.9 ± 2.9
Plasma outcome measures			
Fasting glucose (mmol/L)	5.4 ± 0.1	5.3 ± 0.1	5.5 ± 0.1
Fasting serum insulin (mIU/L)	10.1 (6.1)	10.2 (7.8)	10 (5.8)
HOMA-IR	2.3 (1.4)	2.3 (2.3)	2.3 (1.1)
CRP (mg/L)	2.4 (4.6)	3.2 (4.6)	2.1 (4.9)
GSK-3β (ng/mL)	2.5 (2.1)	2.8 (1.9)	2.1 (2.9)
Red blood cell measures			
Eicosapentaenoic acid ** (%*w/w*)	1.1 (0.4)	1.0(0.4)	1.2(0.4)
Docosahexaenoic acid ** (%*w/w*)	6.1 ± 0.2	6.2 ± 0.3	6.0 ± 0.2
LCn-3UFA (%*w/w*)	7.1 (2.1)	7.0(2.0)	7.1(2.3)
Physical activity * (metabolic equivalents-min/week)	2733 (4422)	2504 (6066)	2768 (3888)

* Baseline physical activity is missing for n = 1 participant. ** Baseline EPA and DHA data are missing for n = 2 participants. Data tested for normality and reported as the mean ± SEM or median (IQR) as appropriate. HOMA-IR: homeostatic model of assessment-insulin resistance. CRP: c-reactive protein. LCn-3PUFA—long-chain omega-3 polyunsaturated fatty acids.

**Table 2:** Changes to the study parameters within and between the placebo and the DHA-rich fish oil groups during the intervention period.

Parameters	Post-Intervention	Change from Baseline	Between Group Differences
			Mean Difference (95% CI)
**Fasting glucose (mmol/L)**			
Normal range: 3.3–5.5 mmol/L			
Placebo	5.2 ± 0.1	−0.04 ± 0.1	
DHA-rich fish oil	5.5 ± 0.1	0.04 ± 0.1	0.1 (−0.1, 0.3)
**Fasting insulin (mIU/L)**			
Normal range: <10 mIU/L			
Placebo	12.0 ±1.1	0.4 ± 0.6	
DHA-rich fish oil	10.0 ± 0.9	−1.3 ± 0.7 *	−1.7 (−3.5, 0.14) **
**HOMA-IR**			
Placebo	2.9 ± 0.3	0.1 ± 0.2	
DHA-rich fish oil	2.4 ± 0.2	−0.3 ± 0.2	−0.4 (−0.9, 0.1) **
**C-reactive protein (mg/L)**			
Normal range: < 3.0 mg/L			
Placebo	4.6 ± 0.9	−0.5 ± 0.4	
DHA-rich fish oil	3.4 ± 0.6	−0.7 ± 0.6	−0.2 (−1.8, 1.3)
**Glycogen synthase kinase-3β (ng/mL)**			
Placebo	2.8 ± 0.4	−0.5 ± 0.4	
DHA-rich fish oil	2.4 ± 0.3	−2.3 ± 0.3 *	−1.8 (−2.7, −0.9) **
**Eicosapentaenoic acid (%*w/w*)**			
Placebo	1.0 ± 0.1	−0.1 ± 0.1	
DHA-rich fish oil	1.8 ± 0.1	0.5 ± 0.1 *	0.6 (0.4, 0.8) **
**Docosahexaenoic acid (%*w/w*)**			
Placebo	6.0 (0.2)	−0.1 (0.9)	
DHA-rich fish oil	9.7 (0.3)	3.5 (1.7) *	3.7 (3.1, 4.3) **
**LCn-3PUFA (%*w/w*)**			
Placebo	7.1 (0.2)	−0.1 (1.04)	
DHA-rich fish oil	11.1 (0.5)	4.1 (2.1) *	4.2 (3.6, 5.0) **

Data are presented as the mean ± SEM, and alpha set at *p* < 0.05. * significant within-group changes from baseline ** between group differences (change from baseline) assessed using regression analysis adjusted for baseline value, and presented as the mean difference (95% CI). HOMA-IR: homeostatic model of assessment-insulin resistance. CRP: c-reactive protein. LCn-3PUFA—long-chain omega-3 polyunsaturated fatty acids.

**Table 3:** Effects of DHA-rich fish oil on blood markers stratified by participants’ baseline inflammation status.

	C-Reactive Protein <2.4 mg/L		C-Reactive Protein ≥2.4 mg/L	
Outcome Measures	Placebo	DHA-Rich Fish Oil	Placebo	DHA-Rich Fish Oil
**Fasting glucose (mmol/L)**				
Baseline	5.2 ± 0.2	5.5 ± 0.1	5.3 ± 0.1	5.4 ± 0.2
Δ Baseline	−0.1 ± 0.1	−0.1 ± 0.1	−0.03 ± 0.1	0.1 ± 0.1
**Fasting insulin (mIU/L)**				
Baseline	9.2 ± 1.2	8.7 ± 0.5	13.6 ± 1.7	14.4 ± 2.5
Δ Baseline	1.8 ± 0.9	−0.6 ± 0.7	−0.8 ± 0.7	−2.1 ± 1.2
**HOMA-IR**				
Baseline	2.2 ± 0.3	2.1 ± 0.1	3.2 ± 0.4	3.5 ± 0.6
Δ Baseline	0.4 ± 0.2	−1.3 ± 0.2	−0.1 ± 0.2	−0.5 ± 0.3
**Glycogen synthase kinase-β (ng/ML)**				
Baseline	2.5 ± 0.5	3.1 ± 0.5	3.4 ± 0.4	2.6 ± 0.4
Δ Baseline	0.18 ± 0.6	−2.7 ± 0.3	−1.1 ± 0.5	−1.9 ± 0.4
**C-reactive protein (mg/L)**				
Baseline	1.3 (0.1)	1.4(0.6)	6(5.2)	6.5(4.9)
Δ Baseline	0 (0.8)	0.1(0.6)	−0.9(2)	−0.8(2)

Data are presented as the mean ± SEM or median (IQR), and alpha set at *p* < 0.05. Δ change in the value from baseline. HOMA-IR: homeostatic model of assessment-insulin resistance.

## Abbreviations

AD	Alzheimer’s disease
BMI	Body mass index
CRP	C-reactive protein
DHA	Docosahexaenoic acid
EPA	Eicosapentaenoic acid
FFM	Fat free mass
HOMA-IR	homeostatic model of assessment-insulin resistance
IPAQ	International physical activity questionnaire
IR	Insulin resistance
LCn-3PUFA	Long-chain omega-3 polyunsaturated fatty acid
T2D	Type 2 diabetes
WC	Waist circumference
